# Synchrony of hand-foot coupled movements: is it attained by mutual feedback entrainment or by independent linkage of each limb to a common rhythm generator?

**DOI:** 10.1186/1471-2202-7-70

**Published:** 2006-10-26

**Authors:** Fausto G Baldissera, Paolo Cavallari, Roberto Esposti

**Affiliations:** 1 Istituto di Fisiologia Umana II, Università degli Studi, via Mangiagalli 32, 20133 Milano, Italy; 2 Dipartimento di Medicina, Chirurgia e Odontoiatria, Ospedale San Paolo, Università degli Studi, via Di Rudinì 8, 20142 Milano, Italy

## Abstract

**Background:**

Synchrony of coupled oscillations of ipsilateral hand and foot may be achieved by controlling the interlimb phase difference through a crossed kinaesthetic feedback between the two limbs, or by an independent linkage of each limb cycle to a common clock signal. These alternative models may be experimentally challenged by comparing the behaviour of the two limbs when they oscillate following an external time giver, either alone or coupled together.

**Results:**

Ten subjects oscillated their right hand and foot both alone and coupled (iso- or antidirectionally), paced by a metronome. Wrist and ankle angular position and Electromyograms (EMG) from the respective flexor and extensor muscles were recorded. Three phase delays were measured: i) the *clk-mov *delay, between the clock (metronome beat) and the oscillation peak; ii) the *neur *(neural) delay, between the clock and the motoneurone excitatory input, as inferred from the EMG onset; and iii) the *mech *(mechanical) delay between the EMG onset and the corresponding point of the limb oscillation. During uncoupled oscillations (0.4 Hz to 3.0 Hz), the *mech *delay increased from -7° to -111° (hand) and from -4° to -83° (foot). In contrast, the *clk-mov *delay remained constant and close to zero in either limb since a progressive advance of the motoneurone activation on the pacing beat (*neur *advance) compensated for the increasing *mech *delay. Adding an inertial load to either extremity induced a frequency dependent increase of the limb mechanical delay that could not be completely compensated by the increase of the neural phase advance, resulting in a frequency dependent increment of *clk-mov *delay of the hampered limb. When limb oscillations were iso- or antidirectionally coupled, either in the loaded or unloaded condition, the three delays did not significantly change with respect to values measured when limbs were moved separately.

**Conclusion:**

The absence of any significant effect of limb coupling on the measured delays suggests that during hand-foot oscillations, both iso- and antidirectionally coupled, each limb is synchronised to the common rhythm generator by a "private" position control, with no need for a crossed feedback interaction between limbs.

## Background

A recent analysis of the voluntary oscillatory movements of one single extremity in different mechanical contexts (foot) [1] (hand) [2] led to the hypothesis that these movements are controlled by a neural mechanism that compares the intended position (encoded by a "central command") with the actual position (encoded by the kinaesthetic afferences). The sign and size of the detected position error would in turn determine the direction (agonist vs antagonist) and the amount of the compensatory motor activation. By this "private" position controller, each limb may overcome the mechanical contingencies by a continuous adjustment of the movement to the central motor command.

This hypothesis has been recently tested by verifying how far subjects could maintain a null position error when the oscillation frequency or the mechanical context was changed. In this aim they were asked to rhythmically oscillate their extremities so that the oscillation peaks were synchronised to the beat of a metronome. The position error was then estimated as the phase delay between the metronome beat (the "intended" oscillation peak) and the actual oscillation peak (Esposti, Cavallari, Baldissera 2006, submitted). With the limbs unloaded, the peak of both the hand and foot oscillations maintained a slight constant phase delay with respect to the metronome signal over the explored frequency range (0.4–3.0 Hz). This constancy was obtained by phase-advancing, at each frequency increment, the EMG activation with respect of the metronome beat by the amount necessary to compensate for the simultaneous increase of the lag between the EMG and the movement, caused by the limb mechanical impedance. After loading of either limb, the increase of the oscillation frequency induced larger EMG-movement delays and the anticipatory compensation, albeit increased, became insufficient, so that the movement progressively phase-lagged the metronome. Finally, the compensatory phase-advance of the muscular activation in both the unloaded or loaded conditions could be accurately simulated by a neural network model that compares a central command (the intended position) to the limb actual position, acting as a closed-loop PID (Proportional, Integrative and Derivative) controller.

The above experimental results and the model simulations strongly suggest that the synchronisation of the rhythmic oscillations of either hand or foot with a time keeper is sustained by a "private" feed-back control that conforms the position of each limb to that encoded in the central voluntary command. If so, when movements of the two limbs are coupled, the position controller of each limb should per se be sufficient to keep the two limbs synchronised with each other (since both are linked to the same external clock) without need for any information exchange between them. This should occur even when the limbs, as in the case of the hand and foot, have different mechanical properties, so that a different frequency-dependent compensation is required for each segment.

With regard to interlimb synchronisation, it was earlier observed that during hand-foot coupled oscillations (from 0.6 up to 3.2 Hz) the interlimb phase difference is kept almost constant by phase-advancing the activation of the hand movers with respect to the foot movers [3]. This finding led us to propose that the control mechanism may: i) compare the kinaesthetic signals from the two moving limbs; ii) compute the instantaneous phase difference between the limbs positions and iii) utilise the resulting phase error for advancing the activation in the lagging limb with respect to the leading limb. Set againts this background and arguing that the kinaesthetic feedback from a moving extremity might operate also when only one extremity is oscillated and the second is still, we investigated the possibility that kinaesthetic afferences from one limb could influence the motor pathways to the other limb [4-7]. Specifically, the excitability of the H-reflex and corticospinal pathways to the resting forearm muscles was tested during cyclic voluntary oscillations of the ipsilateral foot. It was found that excitability in both the descending and reflex motor pathways to wrist movers was sinusoidally modulated during foot oscillations. However, such modulation cannot be related with the kinaesthetic inflow from the foot since, after having artificially modified the EMG-movement phase relations by applying an inertial load to the foot, the modulation remained phase-linked to the activation of foot movers, not to the foot angular position.

In summary, while we found no evidence in favour of a crossed kinaesthetic feedback between the extremities, other clues suggested that each limb is supplied with a control mechanism that compensates for the limb mechanical impedance. In this perspective, synchrony between the hand and foot during coupled oscillations might result from the fact that both segments automatically maintain the synchronism with respect to a common central command, without the need of any inter-limb kinaesthetic feedback. This paper will present further elements in favour of this view.

It might be argued that if the oscillation of each extremity is synchronised to a central rhythm generator by a "private" controller, then the phase relation between the output of the rhythm generator and the movement should be identical when the segment is moved alone and when it is coupled with another extremity. Alternatively, if each segment entrains the oscillations of the other through crossed kinaesthetic afferences, the phase linkage between the generator output and the movement might be different when the segments are moved separately or together. For example, let's suppose that one segment lags the rhythm generator (and the other segment) because of its larger inertial resistance. A feedback mechanism, which monitors the inter-segmental delay by comparing the kinaesthetic afferences from the two limbs, might reduce the inter-limb delay by either anticipating the lagging segment or delaying the leading one: in either case, however, its intervention modifies the segment(s) "intrinsic" delay with respect to the central command.

Assuming that during careful synchronisation of a cyclic movement with a metronome the central voluntary command is phase-locked to the metronome beat, we measured the synchronisation between the limb oscillations and the pacing signal when the two segments were moved in isolation and when the two limbs were coupled, both iso- and antidirectionally. Based on the above considerations, we would expect that the activation of a crossed kinaesthetic feedback during coupling should modify the clock-movement synchronisation by either anticipating the lagging ("heavier") segment or delaying the leading ("lighter") one.

We also examined whether and how the synchronisation is influenced by changing the oscillation frequency (between 0.4 and 3.0 Hz) and by separately loading the hand or the foot, in order to increase the mismatch in their mechanical impedance.

## Results

**Figure 1:**
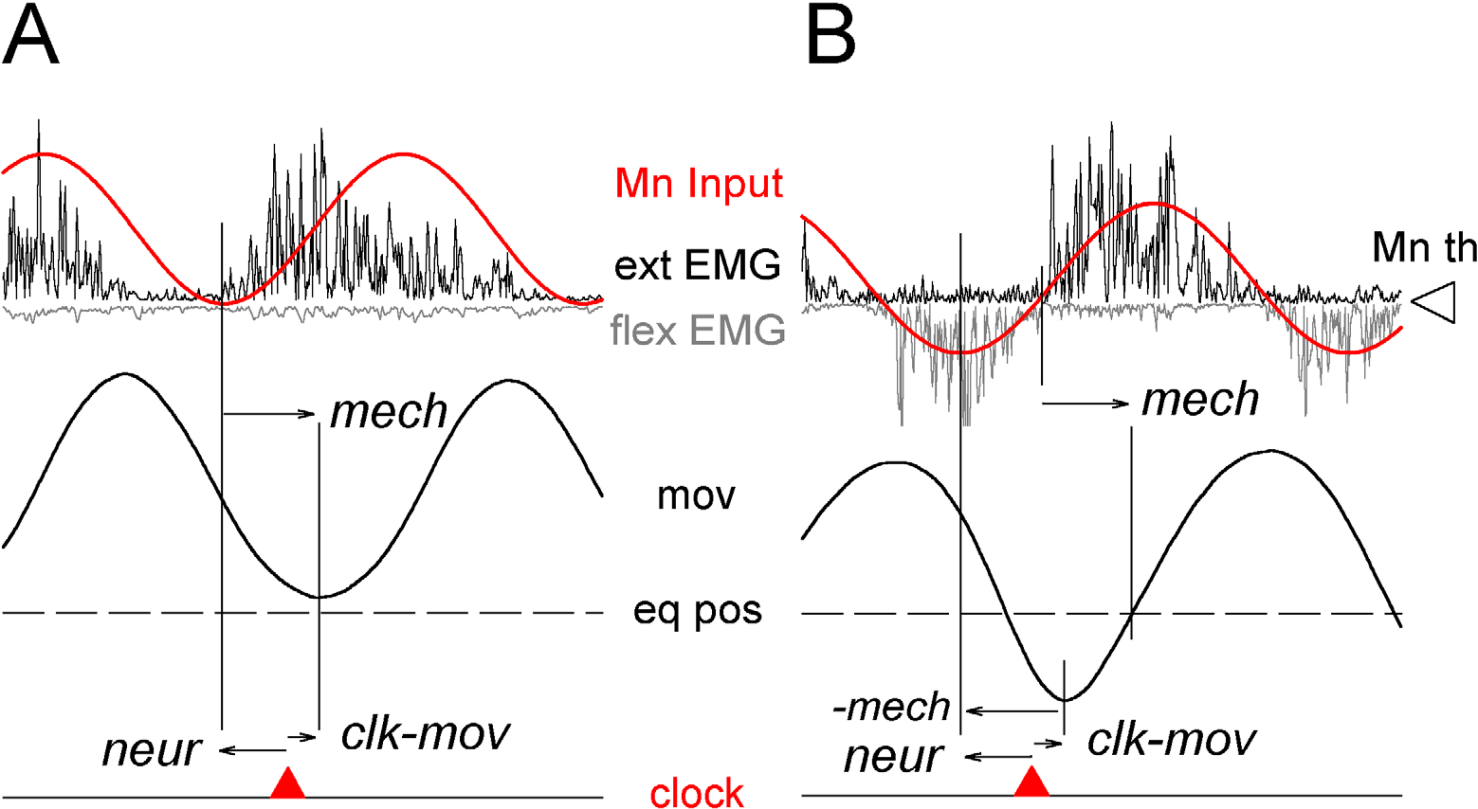
**Schematic representation of the relevant phase measurements**. Measurements are illustrated at a limb oscillation frequency high enough to induce a phase delay between EMG and movement. In **A **and **B**, the *upper traces *describe, superimposed on each other, 1) the rectified EMG from extensors (ext EMG, black trace) and flexors (flex EMG, grey trace, reversed) recorded during a movement cycle and, 2) the sinusoidal input to extensors motoneurones (Mn input, MnI, red line) supposed to drive the limb oscillation. Mn th (open arrowhead) is the motoneurone threshold level. MnI to flexor motoneurones has the same course but is sign-reversed. *Lower traces *describe the ensuing movement (mov), horizontal dashed lines mark the limb passive equilibrium position (eq pos). Upward red arrowheads give timing of the clock signal. In **A **(oscillations all above the equilibrium) the motoneurone input (MnI) is supraliminal in extensor motoneurones (and subliminal in flexor) the whole cycle along. In **B **(oscillations crossing the equilibrium) the MnI is first excitatory in flexor (reversed, as the simultaneous flex EMG burst) and then in extensor motoneurones. The MnI lower peak (in **A**) and the MnI threshold crossing in either antagonist (in **B**) coincide (omitting the small, fixed conduction time) with the onset of the EMG burst in the related muscle. Three phase differences were measured: i) the *clk-mov *delay, between the clock and the oscillation peak; ii) the *mech *delay, between the corresponding points in the MnI (i.e., EMG onset) and movement traces (movement peak in **A**, equilibrium crossing in **B**); iii) the *neur *delay, between the clock and the peak of the motoneurone input. The latter results from the difference (*clk-mov *-*mech*): this is directly visible in **A **and put in evidence in **B **by the -*mech *arrow, connecting the peaks of the MnI and movement sine-waves.

The aim of this investigation was to compare the synchronisation of the hand and foot oscillations with a pacing signal when the two segments were moved separately and when they were iso- or antidirectionally coupled. This was done by measuring the phase delays between the rhythmic clock beat and one peak of the limb oscillation (*clk-mov *delay). The *clk-mov *delay includes a neural delay, between the clock signal and the synaptic input to motoneurones (clk-MnI, or *neur*, delay), plus the lag between the motoneurone input signal and the movement (MnI-mov, or *mech *delay), influenced by the mechanical impedance of the limb. On the assumption that the rhythm generator is synchronised with the clock beat as the task requires, the *neur *delay represents the phase difference between the output of the central rhythm generator and the input to motoneurones. It will be shown that such delay is negative, i.e. a phase advance. The way in which the above delays were estimated is illustrated in Fig. 1 and described in detail in the Methods section.

In different experimental series, the 10 subjects were asked to rhythmically oscillate the two segments both alone and coupled together, first in isodirectional (frequency range 0.4–3.0 Hz) and then in antidirectional association (frequency range 0.4–1.8 Hz). Subjects had to take the maximal care that the peak of the segments' oscillations coincided with the beat of a metronome. All the trials were also repeated after enhancing the mechanical difference between the extremities by loading either the hand or the foot with a rotating mass. The three relevant phase differences (*clk-mov*,*mech *and *neur *delays, see Methods) were measured and plotted against the oscillation frequency.

### Hand and foot unloaded

#### Separate oscillations of the hand and foot and isodirectional hand-foot coupling

**Figure 2:**
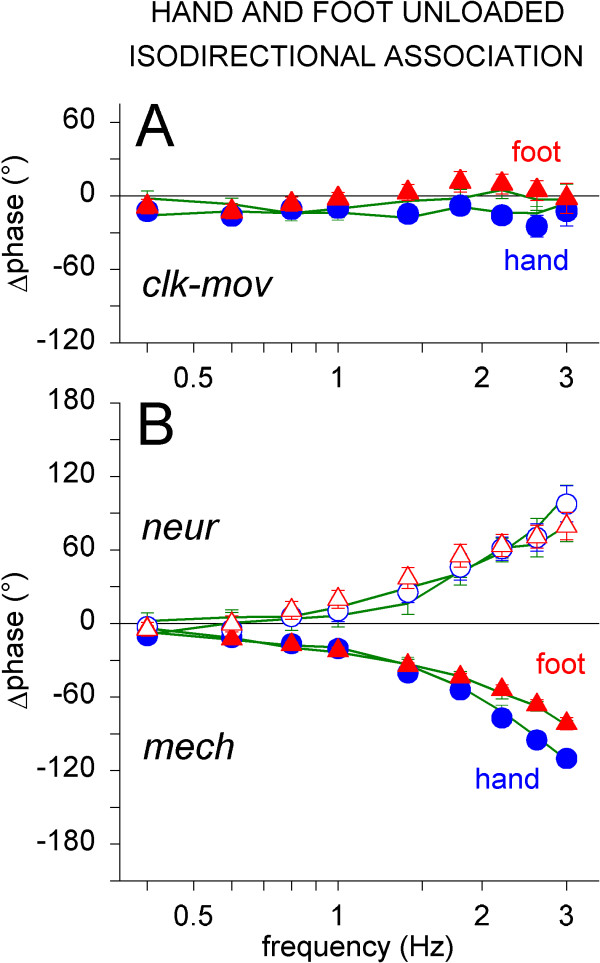
**Synchrony of separate or isodirectionally coupled hand and foot oscillations with the external clock**. Relationships between the three relevant phase delays (*clk-mov*, *mech *and *neur*) and the limbs oscillation frequency (0.4–3.0 Hz). A continuous line connects data from the uncoupled limbs, data from coupled movements are illustrated by symbols (hand, blue circles; foot, red triangles). **A**. In both limbs the *clk-mov *delay, measured between the clock beat and the flexion peak of the oscillations, remained constant over the whole frequency range (no significant effect of frequency). Negative *clk-mov *values when the oscillation peak followed the clock beat. **B**. The *mech *curves (same limb symbols as in **A) **trace the increasing lag of the hand and foot movements with respect to the EMG activation, caused by the segments mechanical impedance. Despite the increasing lag of mechanical origin, in either extremity the *clk-mov *synchrony was constant because of the progressive phase-advance of the motor activation with respect to the clock signal, (*neur *curves, open symbols in **B**). Note that the zero reference for the *mech *curves is the EMG onset while for the *neur *curves it is the clock beat. All curves obtained during isodirectionally coupled hand-foot oscillations virtually overlap the corresponding curves obtained during separate oscillations of the two segments (green lines). Vertical bars in **A **and **B **= Standard Error of the Mean (covered by the symbol in most instances).

Figure 2 reports the phase curves obtained from the hand and the foot both when they were separately moved (continuous lines) and when they were isodirectionally coupled (symbols, hand: blue circles; foot: red triangles)

Two aspects are noteworthy. First, in both extremities, either coupled or uncoupled, the movement peak maintained a constant phase delay from the metronome beat (*clk-mov *curves in plot 2A, negative values when the oscillation peak follows the metronome beat). The way in which this phase lock was achieved is understood by examining the curves in plot 2B. The two *mech *curves describing the mechanical phase-response of each oscillating segment, either alone (continous line) or coupled (symbols, same as in plot 2A), could be well fitted by a pendulum equation (Table 2). Confirming previous observations [3], the resulting resonance frequency (*f*_*r*_) was always significantly lower for the hand than for the foot (p < 0.00001), indicating a relatively larger influence of the moment of inertia in the upper extremity. It is easily argued that if the onset of muscular activation in either segment had maintained a stable phase delay with respect to the metronome beat then the *clk-mov *curve would have run parallel to the decaying *mech *curve. Thus, the observed constancy of the *clk-mov *delay over the entire frequency range in both extremities is necessarily correlated to a progressive phase advance of the motoneurone input on the clock beat (*neur *curves, open symbols in Fig. 2B), an advance which virtually mirrors in either extremity the increasing lag of the corresponding *mech *curves.

The second noteworthy feature of Fig 2 is seen when comparing the *clk-mov, mech *and *neur *phase-relations of each limb, obtained during isodirectional coupling (symbols), with the corresponding relations obtained when movements were performed with the hand or foot alone (solid lines). In either limb, the curves referring to isolated movements are practically superimposed to those referring to coupled movements, indicating that the limbs behave in a virtually identical manner when they move in isolation or coupled.

Statistical comparison was drawn on the *clk-mov *data collected during associated *vs *isolated limb oscillations by a two way (coupling × frequency) repeated measures ANOVA, which failed to uncover any main effect of either coupling or frequency, as well as any interaction (numerical results of the statistical test in Table 1). Comparing the best-fit functions of the *mech *curves through the extra sum-of-squares F test, also showed that limb coupling did not introduce any significant modification in the *mech *curves (numerical results of the statistical test in Table 2).

#### Antidirectional hand-foot coupling

**Figure 3:**
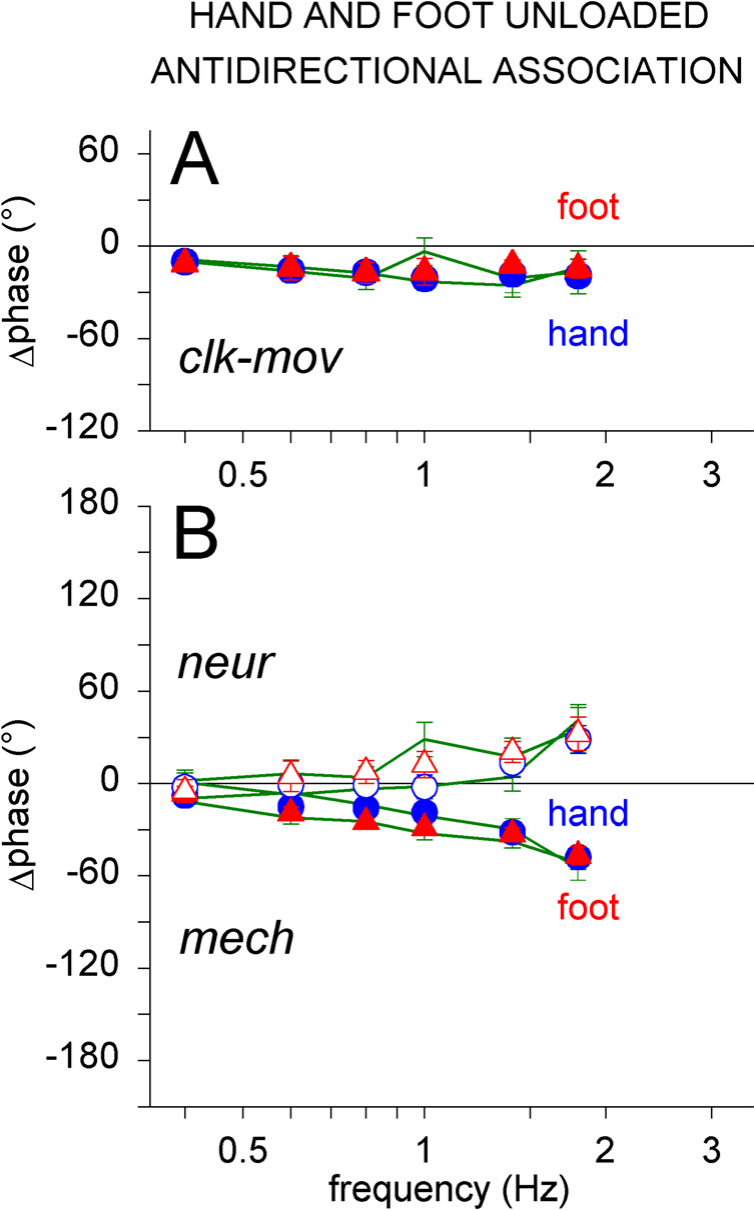
**Synchrony of separate or antidirectionally coupled hand and foot oscillations with the external clock**. **A**. When the hand and the foot were moved either separately or antidirectionally coupled, the *clk-mov *delay remained constant (no significant effect of frequency) as the frequency increased from 0.4 to 1.8 Hz. Same representation and symbols as in Fig. 2. Constancy of the *clk-mov *was achieved despite the increasing lag of the hand and foot movements with respect to the EMG activation (*mech *curves, filled symbols in **B**), thanks to the progressive phase-advance of the motor activation with respect to the clock signal (*neur *curves, open symbols in **B**). All curves obtained during antidirectionally coupled hand-foot oscillations virtually overlap the corresponding curves obtained during separate oscillations of the two segments (green lines). Vertical bars in **A **and **B **= Standard Error of the Mean (covered by the symbol in most instances).

In these trials the explored frequency range ended at 1.8 Hz, the maximal frequency that all subjects were able to follow in antidirectional association. The *clk-mov *curves of Fig. 3A (same symbols as in Fig. 2A) show that the movement peak of either extremity maintains a constant phase delay from the clock in this association too (no significant frequency effect, Table 1).

Due to the 1.8 Hz frequency limit, the two *mech *curves (Fig. 3B, same symbols as in Fig. 2B) only included the flat initial portion of the decay. Consequently, fitting with the pendulum equation led to poorer R^2 ^values and larger variability of the estimated parameters (Table 2) than in trials when frequency was increased up to 3 Hz (limbs alone or isodirectionally coupled). Since this would weaken the statistical comparison between the best-fit curves, the *mech *data for the coupled and uncoupled conditions were also matched by a two way (coupling × frequency) ANOVA, which confirmed the presence of a significant frequency-effect (p < 0.0000001 for either limb), in the absence of a main effect of coupling and of any frequency-coupling interaction (p always > 0.65).

As seen for the isodirectional association, a progressive phase advance of the motor input on the clock beat (*neur *curves, open symbols in Fig. 3B) virtually mirrored in either extremity the increasing lag of the corresponding *mech *curves.

Fig. 3 also allows the direct comparison between the curves referring to anti-phase coupling (symbols) and the corresponding curves (solid lines) obtained when the same subjects performed oscillatory movements with either the hand alone or the foot alone. Inspection of the curves suggests that both limbs behave in a virtually identical manner when they move in isolation or coupled. This was confirmed by statistical analysis (Tables 1 and 2), which did not uncover any significant difference.

In conclusion, the striking similarity between the curves of the coupled and uncoupled limbs strongly supports the view that one and the same mechanism operates in both instances. It might therefore be argued that the "private" controller, which compensates for the phase lag of each extremity when it is moved alone, is also sufficient to keep synchrony between hand-foot coupled oscillations driven by a common clock signal, without need for any crossed feedback interaction between the two limbs.

However, the lack of any significant change in synchronisation when the limbs were moved separately or together, might have a more trivial explanation, i.e. that the difference between the limbs mechanical properties were significant but minor, perhaps too small to elicit any sign of interaction between the two moving segments. The mechanical difference between hand and foot were therefore experimentally enhanced by separately adding an inertial load to one or the other extremity (see Methods).

### Inertial loading of the limbs

Results of the loading experiments will be illustrated following the same criteria used in Figs. 2 and 3 for the unloaded condition (solid lines, curves obtained during isolated oscillations; symbols, curves obtained during coupled oscillations). Inertial loading of the cyclic movements of the hand or foot was obtained by fixing a metal disk concentrically to the axis of rotation of the respective platform (see Methods). The moment of inertia of each disk was about 3.5 times that of the related limb plus the rotating platform.

### Hand loading

#### Separate hand-foot oscillations and isodirectional coupling

**Figure 4:**
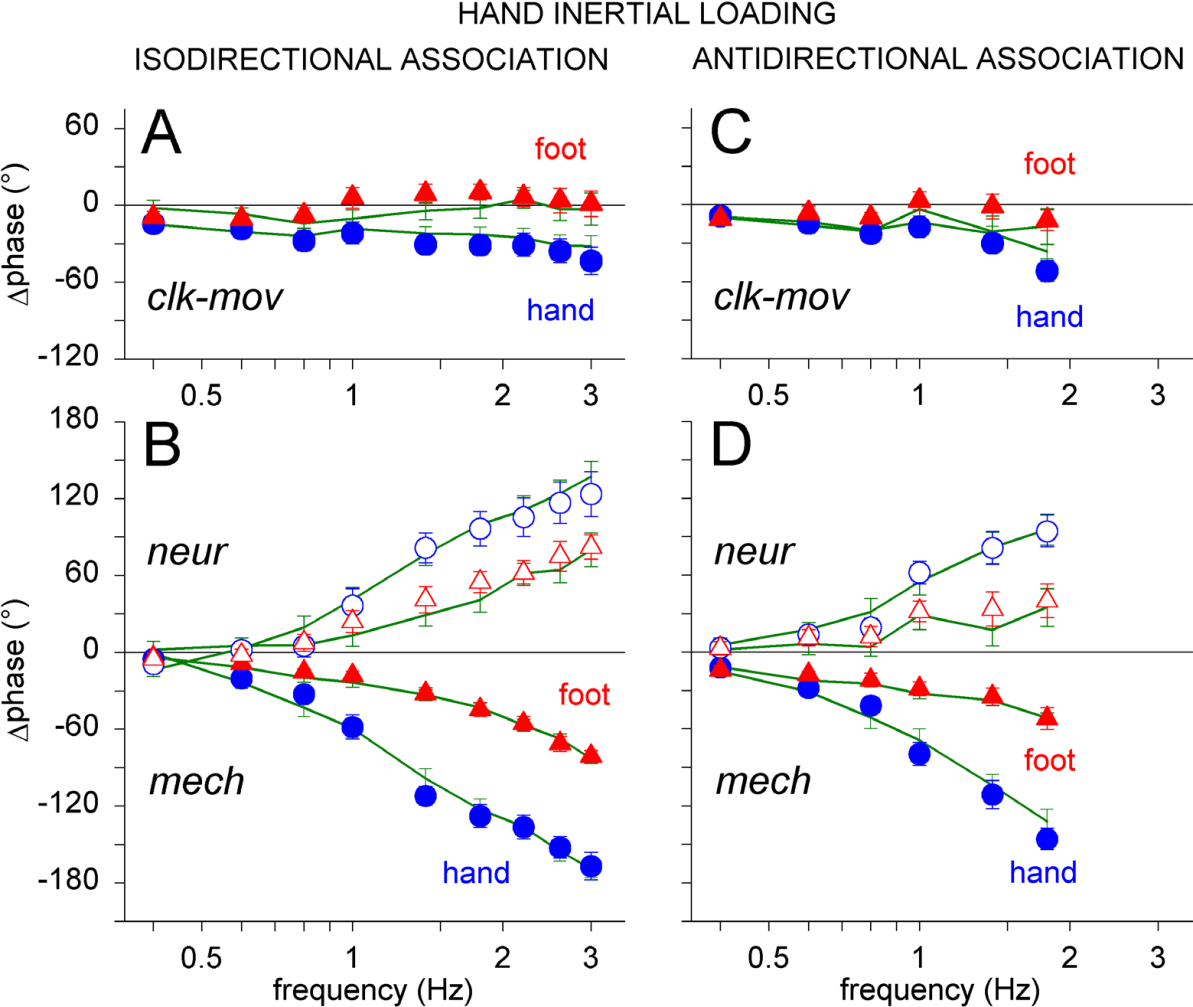
**Effect of hand inertial loading on limb synchrony with the external clock**. The plots illustrate data obtained during isodirectional (**A-B**) and antidirectional association (**C-D**), with the same limb symbols as in Figs. 2 and 3. On each plot, solid green lines connect data points obtained during uncoupled oscillations while symbols refer to coupled oscillations data. Hand loading increased the hand movement delay with respect to the clock (*clk-mov *curve, filled circles in **A **and **C**) in a frequency-dependent manner. This reflected the large increase of the hand mechanical impedance, which caused a steep decay of the *mech *curve (filled circles in **B **and **D**) that was not fully compensated by the advance of motor activation (*neur *curve, open circles in **B **and **D**). Foot curves were unchanged. Note that in either association direction, curves obtained during coupled movements fully overlap those obtained when the hand and foot were moved separately.

Loading produced an earlier decay of the hand *mech *curve, with a satisfactory fitting by the pendulum equation and a conspicuous decrease of the hand *f*_*r *_(Table 2), thus strongly increasing the difference between the hand and foot mechanical responses (Fig. 4B). Correspondingly, a clear-cut increase in the slope of the hand *neur *curve developed symmetrically to the decay of the *mech *curve. The compensation, however, was not as effective as in the unloaded condition and the *clk-mov *delay for the hand significantly increased when the frequency increased (Fig. 4A and Table 1, main effect of frequency).

Loading, however, did not introduce any significant difference between curves obtained during uncoupled and isodirectionally coupled movements of the hand (compare symbols and solid lines in Fig. 4A–B, see Tables 1 and 2 for statistical comparisons).

#### Antidirectional coupling

As in the case for isodirectional coupling, loading produced the earlier decay of the hand *mech *curve (Fig. 4D, blue filled circles) and a symmetrical, clear-cut increase in the slope of the *neur *curve (blue open circles). The advance in motor activation, however, was not as effective as in the unloaded condition in compensating for the *mech *delay, thus leading to a significant increase of the hand *clk-mov *delay when the frequency increased (Fig. 4C filled circles, Table 1, main effect of frequency). Again, no difference was detectable between the curves obtained during uncoupled and antidirectionally coupled hand-foot oscillations (compare symbols and solid lines in Fig. 4C–D, see Tables 1 and 2 for statistical comparisons).

Finally, note also that, in both iso- and antidirectional associations, the curves measured in the unloaded foot did not differ from those measured when the foot was moved alone or was coupled with the unloaded hand (Tables 1 and 2).

### Foot loading

#### Separate hand-foot oscillations and isodirectional coupling

**Figure 5:**
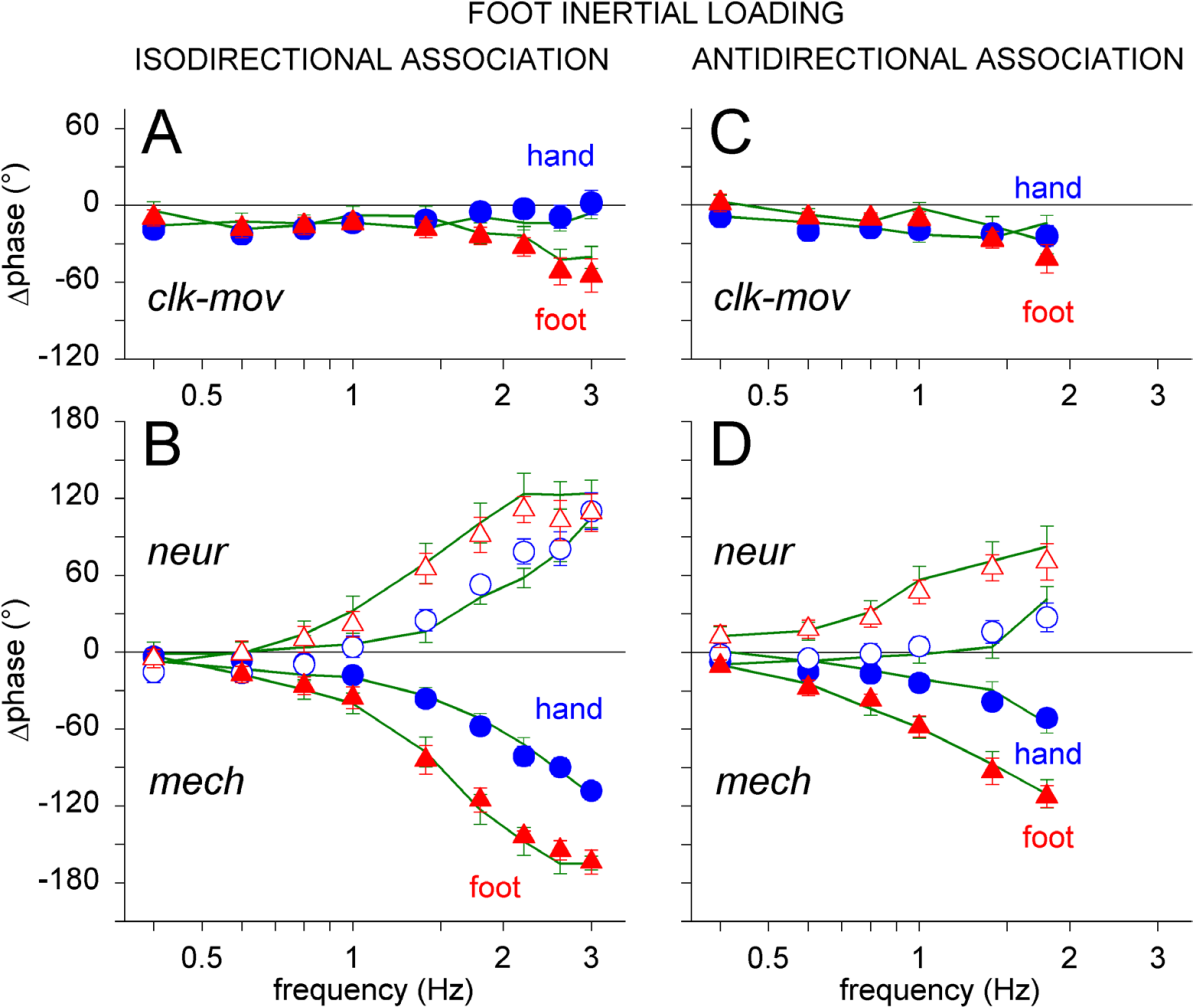
**Effect of foot inertial loading on limb synchrony with the external clock**. Data obtained during isodirectional (**A-B**) and antidirectional association (**C-D**) are illustrated with the same symbols as is Figs. 2 and 3. Foot loading increased the foot movement delay with respect to the clock (*clk-mov *curve, filled triangles in **A **and **C**) in a frequency-dependent manner because of the large increase of the foot mechanical impedance (*mech *curves, filled triangles in **B **and **D**), which was not fully compensated by the advance of motor activation (*neur *curve, open triangles in **B **and **D**). Curves of the hand remained unchanged. Note that in either association direction, curves obtained during coupled movements (symbols) fully overlap those obtained when the hand and foot were moved separately (green lines).

After applying an inertial load to the foot platform, the foot *mech *response decayed faster than that of the unloaded hand (compare filled circles with filled triangles in plot 5B). The reactive anticipation of the motor output to foot movers (*neur *curve, open symbols in 5B) was enhanced with respect to the unloaded condition but the advance was apparently insufficient to maintain the clock-movement synchrony over the entire frequency range, as witnessed by the progressive increase of the *clk-mov *delay over 1.5 Hz (filled triangles in Fig. 5A) resulting in a significant frequency-dependence of the *clk-mov *curve (Table 1).

Again, no significant difference (Tables 1 and 2) was visible between the curves obtained during the uncoupled (solid lines) or the coupled movements (symbols).

#### Antidirectional coupling

Foot loading produced a faster decay of the *mech *phase-response (Fig. 5D, filled triangles) and an enhancement of the *neur *anticipation (open triangles) also during antidirectional association. Again, the *neur *compensation was insufficient to maintain the clock-movement synchrony so that the *clk-mov *delay (filled triangles in Fig. 5C) increased in a frequency-dependent manner (Table 1). Once more, however, no difference was visible between the curves obtained during the uncoupled (solid lines) or the coupled movements (symbols) (see statistical details in Tables 1 and 2).

Finally, in neither iso- nor in antidirectional association, did loading of the foot introduce any significant change in the curves of the coupled unloaded hand, which remained indistinguishable from the corresponding curves obtained when both limbs were unloaded or the hand was moved alone.

#### Interlimb phase relations: effects of loading and association direction

**Figure 6:**
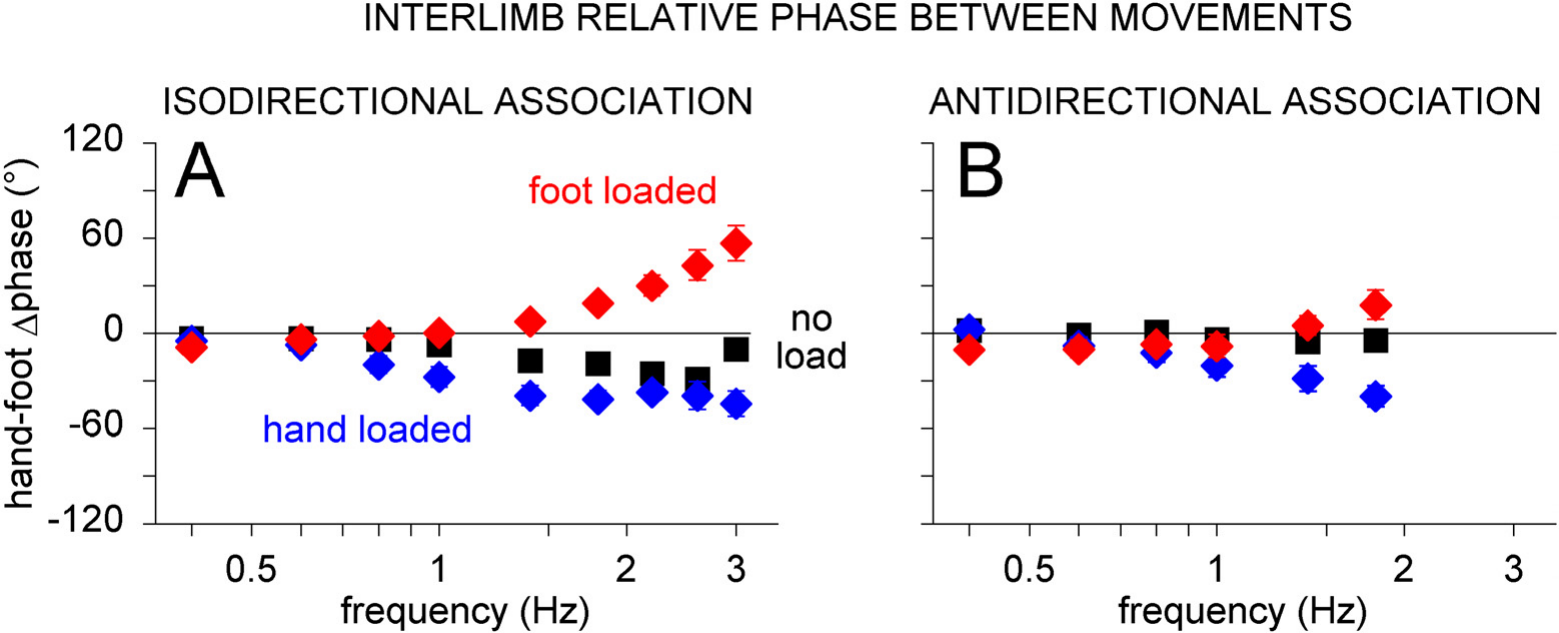
**Inter-limb phase differences between hand and foot movements**. Inter-limb phase differences obtained in different loading conditions, as indicated by labels, and in the two association directions (**A **isodirectional; **B **antidirectional). Positive values on ordinate indicate foot delays, negative values indicate hand delays. When both limbs were unloaded (black squares) the hand lagged the foot by a mean value of 13.5° during iso- and 2.7° during antidirectonal association. Loading the hand increased the mean hand-foot lag and made it dependent on frequency (blue diamonds). Reciprocal changes occurred after loading the foot (red diamonds).

The commonest way to describe associations of cyclic limb movements is plotting their inter-limb phase relations. From this material such relations were easily obtained as the differences between the *clk-mov *curves of the hand and foot for each of the various loading and coupling combinations. These interlimb relations are shown in Fig. 6 for isodirectional (A) and antidirectional (B) oscillations, with different symbols for the three loading conditions (black squares: both segments unloaded; blue diamonds: hand loaded, red diamonds: foot loaded). The illustrated relations confirm the previously described delaying effects of inertial loading on the hand curves [8] and add the description of the effects of foot loading on the curves.

Statistical evaluations were performed on the data included in the frequency range common to both iso and antidirectional association, *i.e*., between 0.4 and 1.8 Hz. A 3 way repeated measures ANOVA (frequency × loading × association direction) showed significant main effects of loading (F_(2,16) _= 12.83 *p *< 0.0005) and frequency (F_(5,40) _= 5.70 *p *< 0.0005) and a significant load × frequency interaction (F_(10,80) _= 22.04 p < 0.000001). In turn, the Neumann-Keuls post-hoc test on the interaction disclosed that the frequency effect was significant when either limb was loaded but not when both limbs were unloaded. In contrast, no difference, was found between curves obtained in iso- and antidirectional association (no main effect of association and no interaction with frequency and/or loading, *p *always >0.25).

## Discussion

The present results show that the synchrony between the cyclic movements of the hand or foot with respect to an external time-giver is unchanged when the two extremities are oscillated separately and when they are coupled, both isodirectionally (in-phase) and antidirectionally (anti-phase).

The virtually identical hand/foot synchronisation with the clock beat, irrespectively of the fact that the segments were moved alone or together, would indicate that a cross-talk between the moving limbs is unnecessary to guarantee the coupling control, even when the mechanical disparity between the moving segments is heavily enhanced. As a matter of fact, loading one limb induced a progressive phase lag of its movement with respect of the clock signal, but this lag did not change significantly between the "alone" and "coupled" conditions. Since also the synchronisation of the second (unloaded) limb remained indistinguishable either when coupled or moved alone, it may be concluded that no significant adjustment occurred after coupling in either the unloaded or the loaded segments. Consequently, the effects induced by loading on one extremity were directly transferred into commensurate phase shifts between the two. Accordingly, it may be reasonably suggested that hand-foot synchronisation during coupling results from the independent linkage of each segment with the clock signal, through a "private" feedback position control based on the kinaesthetic afferences from the same segment. Then, the inter-limb phase relation merely results from the algebraic summation of the phase delay that each limb has with respect to the reference signal when it is moved alone.

It is well known that when coupling segments of the upper and lower limb on the same side, the preferential association is to move the segments in the same direction [9-14]. Coupling the limbs in phase opposition requires a great attentive effort and spontaneous reversal to in-phase association becomes unavoidable when the oscillation frequency increases, consistently reducing the maximum frequency of the anti-phase *vs *in-phase oscillations.

The present results do not give any apparent indication that the feedback position control considered here may exert differential action during one or the other type of association. Indeed, no significant difference was found between any of the phase curves obtained in the different associations. Intervention of the feedback position control in determining the inter-limb phase relation is apparently straightforward during isodirectional hand-foot movements. It might be argued that a cyclic command is produced by a rhythm generator (synchronised with the external clock) and dispatched in parallel to the motor pathways of the two oscillating segments. In turn, the motor pathway to each segment is provided with a "private" kinaesthetic feedback control that continuously conforms the segment position to the position encoded in the central motor command [2]. In addition, we recently found evidence that the voluntary command producing the oscillation of one extremity, while the other is still, is accompanied by the collateral excitation of the motor pathways to those muscles of the second limb that would produce "isodirectional" movements. This excitation is revealed as a subliminal excitability modulation of the motor pathways when posture is stable [4,5,7,15] and as an overt muscle contraction, featured as an anticipatory postural adjustment (APA) [16], when postural stability is decreased. Thus, when the two extremities are moved together, these collateral postural effects would reinforce the explicit voluntary activation of "isodirectional" muscles.

The present results further show that the "private" position-control of each limb is active also during the anti-phase association, allowing synchronisation with the clock beat up to the maximum frequency (1.8–2.2 Hz in our subjects) allowed to these movements. Considering that during both uncoupled and isodirectionally coupled limb oscillations the synchronisation is maintained up to frequencies well above the latter critical value, it may be argued that the capabilities of the position control will not be saturated during anti-phase movements. This would suggest that the frequency limits of the anti-phase performances, signed by the spontaneous transition from anti- to isodirectional association, should not be dependent on failures of the limb feedback position control based on kinaethetic afferences. This view seems confirmed by Spencer et al. [17] who compared normal subjects and patients with uni- or bilateral deafferentation drawing symmetrical or anti-symmetrical circles with both hands. They found that spatial consistency and position both between and within limbs were disrupted in the absence of somatosensory feedback, whereas this loss was not critical for achieving temporal coupling between the hands nor did it contribute significantly to the disruption of anti-symmetrical coordination at faster movement rates.

It may be worth adding that a possible origin of the spontaneous transitions may instead be the conflict between the voluntary commands and the APAs described above, which are distributed as to facilitate the isodirectional and hinder the antidirectional association. In line with this interpretation, Welsh et al. [18] found that the non-preferential coupling of upper limbs is favoured by increasing the trunk postural stability, i.e. attenuating the need of postural adjustments.

As discussed in Baldissera and Esposti 2005 [16], when performing hand-foot antidirectional movements, which require activating the "isodirectional" muscles in phase opposition, the APAs have to be suppressed or overwhelmed by the voluntary commands. Theoretically, during anti-phase association two commands opposite in phase may be simultaneously supplied to the motor pathways of the two limbs either by activating a second generator that pulsates in phase opposition with respect to the first one or, alternatively, by phase-reversing the motor command from a common time-giver to one of the two segments. The supplement of neural activation required for operating either type of phase-reversal and for contrasting APAs might explain the attentional effort requested when performing antidirectional movements. In turn, the saturation of the effort capabilities when the movement frequency increases or their attenuation by "fatigue" when the exercise is prolonged might explain the spontaneous reversal into the less demanding isodirectional mode. Whatever the mechanism, this may be the level at which different factors act to induce a phase transition from anti- to isodirectional association. In this view, the transition should be caused by a failure in the mechanism that builds up the commands shape, not in the mechanism that controls the accuracy of the limb position with respect to the commands.

Distinction of two control levels for limb movement coupling has already been proposed in the literature. In 1979, Schmidt et al. [19] found that during simultaneous reaching movements there is a strong correlation in time between the two hands, while spatial errors in hitting the targets are poorly correlated. Those authors then proposed that the control of isomorphic movements of two limbs is obtained by one motor program, in which the temporal aspects are specified by parameters common to both movements, while force is coded by other, segment-specific parameters. In turn, Wing and Kristfferson [20] dissected the variance of the inter-tap interval into a *clock *and a *motor *components and Turvey et al. [21] utilised this same technique for identifying two levels of control of bilateral wrist-pendulum coupled oscillations, a *time keeping *and a *motor *function respectively. These last authors observed that, when the mechanical properties of the oscillating system of one side were modified, only the correlation between the right and left *motor *variances was affected while the two *clock *variances were still correlated. Conversely, the contrast of phase symmetry (iso- vs antidirectional oscillations) affected the correlation between the *clock *variances but did not affect right and left *motor *variances [22]. On this basis they concluded that "absolute coordination is to be understood as the assembling of a single common clocking process and not as the linking of already established clocks". Evidence in favour of a central temporal coupling was also found by Semjen and Summers [23] when studying synchronic and alternate bimanual tapping.

The position control proposed here, which conforms the actual to the intended limb position through a peripheral feedback mechanism, seems to fit, both in topology and function, with the *motor *level of the Turvey's reference frame, which is not involved in phase transitions. Indeed, the position control is affected by loading of the limbs while it operates equally well during iso- and antidirectional coupling and isolated movements too.

A subdivision in two hierarchical levels has also been proposed for the cat spinal Central Pattern Generator (CPG) [24,25] that controls locomotion. It was suggested that separate structures within the CPG would provide on the one hand the rhythm generation, on the other the pattern formation, i.e., shaping the final outflow to motoneurones according to the different tasks and contexts, under the influence of peripheral afferences (see also) [26].

The role of one main component of the kinaesthetic input (muscle spindles afferents) in controlling ipsilateral limb movements and coupling between limbs was investigated by Verschueren et al. [27]. Those authors found that tendon vibration of biceps and deltoid muscles perturbs circle drawing with the ipsilateral arm (changing the shape from a circle to an inclined ellipse) but had no effect on drawing with the contralateral, non-vibrated limb. Moreover, the ipsilateral alterations were not different when the contralateral arm was moving or resting. On this basis, they concluded that proprioception exerts an independent *spatial *control on each arm, without any crossed effects. This would support our hypothesis of a "private" position control for each limb. However, since the vibratory stimuli produced a change in the interlimb phase relations, those authors proposed that the *temporal *control between the two arms is dependent on the contribution of proprioception, a conclusion that would sound in contrast with our claim for an independent phase control of each limb. It should be noted, however, that the trajectory disparity resulting from unilateral vibration (elliptical on the vibrated side *vs *circular on the unaffected side) is automatically reflected into a change in the relative phase between the two arms. Thus the change in relative phase that was described as failure in the *temporal *control is seemingly implicit in the unilateral change of *spatial *control. Viewed in this perspective, the contrast with our conclusions would disappear.

With regard to the effects of limb loading on movement coupling, our results replicate those already presented for hand loading [8,10] and supplement them with a description of the effects produced by loading the foot. The observed shifts in the hand-foot relative phase induced by loading substantially conform to those described for asymmetric loading of arm-leg [12] or bimanual [28] oscillations.

Finally, it should be stressed that the present results do not exclude the existence of mutual kinaesthetic influences from one limb to the motor pathways to the other but only demonstrate that these appear not to have any significant influence in synchronising the hand and foot during coupled voluntary movements. Inter-limb reflexes have been repeatedly demonstrated and considered important for ensuring arm-leg coordination during rhythmic locomotor tasks (see for example) [29,30]. However, their features do not seem well suited for sustaining the on-line feedback control of the inter-limb phase difference. Some reflexes are evoked by cutaneous afferences, i.e., poor candidates for precisely monitoring the limb position. Further, their latency appears rather long (80–120 ms, up to one third of the cycle period at 3 Hz) for playing a crucial role in the hypothesised feed-back loop. Finally, since these reflexes are modulated during the locomotion cycle they should interact with the motor output stage of the spinal CPG (cfr.) [29,30], and the latter should be for the most part different from the motor stage of the encephalic rhythm generator that drives the non-locomotor voluntary movements we studied. A separation between the two motor stages is also suggested by the relatively few varieties of locomotory patterns as opposed to the great number of different "focal" voluntary movements. Altogether, these diversities might explain why crossed inter-limb effects are present during locomotion and absent in our context.

Nor do the present findings exclude the possibility that afferent signals elicited by passive oscillations of one limb may draw the central rhythm generator and entrain the movement of another limb, as other periodical signals also do (e.g. the metronome beat). This entraining action on the *time keeper *level might explain results obtained when manipulating kinaesthetic afferences originated outside the moving limbs. For instance, Serrien et al. [31] found that the coordination of homolateral effectors (right arm/right leg) is perturbed by the passive cyclic movements of a third limb, the anti-phase to a larger extent than the in-phase coupling. In turn, Carson et al. [32] observed that passive flexion extension of the left hand, phase-locked to a visual sinusoidal stimulus, favours 90° coupling of similar movements of the right hand with the visual signal.

## Conclusion

The present study provides evidence suggesting that synchrony of coupled hand-foot movements is attained by two independent position controllers, one for each limb, that keep the limb movement synchronous to a common rhythm generator, up to 3 Hz. Because of this mechanism, the inter-limb phase difference results from the algebraic summation of the phase delay that each limb has with respect of the central clock. This apparently applies equally well to the in-phase and anti-phase coupled oscillations.

## Methods

### Experimental procedures

Experiments, approved by the local ethical committee in accordance with the ethical standards of the 1964 Declaration of Helsinki, were performed on 10 subjects of either gender, aged 22 to 66, with no history of neurological disease, who gave informed consent for the study. Subjects were tested on the right side. During the experimental session, they were sitting on an armchair with their right arm at their side, elbow at 90° and forearm fixed to a horizontal arm rest by Velcro straps. The hand was placed prone on a platform pivoting on ball bearings around the wrist joint axis, as to permit vertical hand oscillations in the parasagittal plane. The thigh was horizontal, the leg vertical and the foot placed on a platform pivoting in the parasagittal plane around the ankle joint axis. In part of the experiments, inertial loading of the cyclic movements of the hand or foot was obtained by fixing a metal disk concentrically to the axis of rotation of the respective platform (moment of inertia 15 g·m^2 ^for the hand and 98 g·m^2 ^for the foot). The moment of each disk was about 3.5 times that of the related limb plus the rotating platform and produced a consistent EMG-movement phase shift. Subjects were asked to oscillate their hand and foot, in different combinations (see below), following the rhythm of an electronic metronome.

### Data recording

The ankle and wrist angles were measured by precision potentiometers (Bourns, mod. 6639, 1kΩ) coaxially linked to the platforms' pivots and supplied with 5 V ripple-free DC current. Before each experimental session the transducer reading was calibrated by relating three fixed known angles imposed to the platforms (-90°, 0°, 90°) to the corresponding values of the position signal. Electromyograms (EMG) were recorded through bipolar surface gold-plated electrodes, placed on the belly of Extensor Carpi Radialis (ECR), Flexor Carpi Radialis (FCR) Tibialis Anterior (TA) and Soleus (Sol) muscles, amplified (5–10 k) and band-pass filtered (10–1000 Hz). The metronome output, the goniometric and EMG signals were monitored on an oscilloscope, digitised (National Instruments PCI MIO 16-E4, sampling rate of 2.5 kHz per channel, 12bit resolution) and stored for further analysis.

### Estimate of the equilibrium position

At the beginning of each experiment the hand and foot equilibrium position was measured in each subject. With the subject's forearm muscles fully relaxed (no EMG activity), the experimenter moved the subject's hand to both ends of its movement range around the wrist joint and allowed it to return to equilibrium in two different ways, in order to test for possible friction effects for both slow and fast returns. In 3 trials the hand was left free and reached equilibrium after a few damped oscillations; in 3 other trials the passive hand recoil was antagonised by the experimenter, who limited the recoil speed to less than 1°·s^-1^, monitored on an oscilloscope. The same was done for the ankle joint. In both joints, the final position did not differ more than 2° among the 6 trials. The mean of the six values was used for calculations. The procedure was repeated 3–4 times throughout the experimental session to verify that the equilibrium position had not changed above the variability observed initially.

### Trials design

Subjects were asked to perform cyclic flexion-extensions of the right hand and/or foot and were invited to pay great attention to synchronising their movements with the beat of the metronome. In general, synchronisation might be measured with either the upper or the lower peak of the oscillations (up-beat *vs *down-beat synchronisation) depending on the coupling mode. All subjects followed the down beat synchronisation mode during isolated and isodirectionally coupled oscillations and the results from the two coupling conditions could then be compared with each other. During antidirectional association, instead, the subjects necessarily followed opposite synchronisation modes in the hand and foot. Therefore series of isolated movements of one limb had to be repeated in the up-beat synchronisation mode in order to congruously match with movements of the same limb when it was coupled. This limb could be either the hand or the foot, according to each subject's preference. This procedure led to two different data samples for the limbs isolated oscillations, each one serving as a reference for isodirectional and antidirectional association respectively.

Frequency of uncoupled and isodirectionally coupled oscillations was paced at 0.4, 0.6, 0.8, 1.0, 1.4, 1.8, 2.2, 2.6, 3.0 Hz in random order. Frequencies of antidirectional oscillations did not exceed 1.8 Hz. Every trial included at least 10 cycles. The following combinations were tested: isolated and coupled oscillations with the hand and foot unloaded; 2) isolated and coupled oscillations with the hand loaded and foot unloaded; and 3) isolated and coupled oscillations with the hand unloaded and foot loaded. Conditions that were present in more than one experiment (e.g. the trials "hand unloaded alone" belonging to experiments 1 and 2) were not replicated.

In each trial, the movement amplitude was freely chosen by each subject. It varied between 90° ± 26° at 0.4 Hz and 54° ± 22° at 3 Hz for the hand and between 57° ± 12° at 0.4 Hz and 42° ± 14° at 3 Hz for the foot (mean ± SD).

### Measurements of the relevant phase delays

The aim of this investigation was to compare the synchronisation of the hand and foot oscillations with the pacing signal when the two segments were moved separately and when they were iso- or antidirectionally coupled. This was done by measuring the time interval between the rhythmic clock beat and the chosen peak (see the *Trials design*) of the oscillation (*clk-mov *delay). The *clk-mov *delay includes a neural delay, between the clock signal and the synaptic input to motoneurones (*clk-MnI*, or *neur*, delay), plus the lag between the motoneurone input command and the movement (*MnI-mov*, or *mech *delay) that reflects the mechanical impedance of the limb. These time intervals were then converted into phase delays by normalisation to the cycle duration. In the assumption that the rhythm generator is synchronised with the clock beat, as the task requires, the *neur *delay represents the phase difference between the output of the central rhythm generator and the input to motoneurones. It will be shown that such delay is negative, i.e. a phase advance. It was therefore of interest to separate the *neur *and *mech *components.

In the range of frequencies explored here, the relation between motoneurone input current and isometric force developed by the motor unit is approximately linear [33]. Thus, given a linear force-movement relation [34-39], it may be conceived that sinusoidal limb oscillations are driven by a motoneurone input (synaptic current) of sinusoidal shape, opposite in sign in the two antagonists [1,2]. The entire profile of the MnI cannot be deduced from the EMG recordings since EMG grows faster than the MnI [39,40] because of the dynamic sensitivity of motoneurones [41,42] but its supposed time course can be estimated as that of the sinusoid interpolating the onset of the successive EMG bursts in the antagonists (taking into account that the EMG onset lags the MnI threshold crossing by the negligible fixed time of peripheral conduction).

On these premises, a correspondence between the MnI and the movement can be established at discrete points of the cycle [1,2]. Briefly, at very low frequency, when inertial and viscous resistance is negligible, the MnI and the movement sine-waves will be isochronous. In this condition, if a limb oscillates all above its passive equilibrium position, only one muscle is active throughout the cycle, alternatively getting the limb away from or closer to the equilibrium position. Correspondingly, the input to its motoneurones and the related EMG start growing at the beginning of one movement phase, i.e., in correspondence to one oscillation peak (Fig. 1A). If, instead, the limb oscillates across the joint equilibrium position, conservative forces vanish when equilibrium is reached and muscular intervention is required for continuing the movement. In this second case the MnI threshold crossing (and the EMG onset) in each antagonist corresponds to the instant when the movement crosses the equilibrium (Fig. 1B). In conclusion, whatever the movement range, in near-static conditions a correspondence is established between two related events: i) a definite instant of the MnI (its lower peak or the threshold crossing) and ii) the moment at which the limb distance from the equilibrium position starts increasing. Neglecting the small, fixed conduction delay, EMG and MnI were considered to coincide and the relevant instants of the MnI were evaluated from the EMG recordings. The *mech *delay was thus measured between the EMG onset and the corresponding point of the movement cycle (oscillation peak or equilibrium crossing). In this way, the *mech *data faithfully describe the limb phase response (see next paragraph) and can be used for estimating the values of the limbs mechanical parameters. Accordingly, the neural component (*neur *delay) of the total delay is expressed by the difference *clk-mov *– *mech = neur*. As stated above, it includes the small, fixed delay due to peripheral conduction.

When the oscillation frequency increases, the *mech *delay increases because of the limb impedance and can be measured between the EMG onset and either the oscillation peak or the equilibrium crossing, as specified above (Fig. 1A and 1B). The *neur *delay is again obtained by difference, *clk-mov *– *mech*. In both extremities, the above reference points (clock signal, movement peak, crossing of the equilibrium position and EMG onset) were automatically detected for each movement cycle. An iterative local minimum algorithm detected the movement peak. The onset of each EMG burst was determined by automatic selection of the points where the rectified and integrated (τ = 20 ms) EMG signal crossed a threshold voltage (3 Standard Deviations of the baseline activity) and stayed above it for at least one third of the cycle period. This procedure was followed by visual check for possible errors that were manually corrected on interactive graphics. Finally time intervals were converted into phase delays. In each trial, the phase values measured over 10 successive cycles were averaged. The resulting values were utilised for statistical elaboration (see below) and plotted against the oscillation frequency (*clk-mov, mech *and *neur *curves).

### Mechanical model for limb oscillations

The input-output phase-relation of the prone hand or foot during voluntary oscillations was compared to that of a second order rotating pendulum [34-39], described by the general equation:

T(t)=kθ(t)+ηdθ(t)dt+Id2θ(t)dt2     (1)
 MathType@MTEF@5@5@+=feaafiart1ev1aaatCvAUfKttLearuWrP9MDH5MBPbIqV92AaeXatLxBI9gBaebbnrfifHhDYfgasaacH8akY=wiFfYdH8Gipec8Eeeu0xXdbba9frFj0=OqFfea0dXdd9vqai=hGuQ8kuc9pgc9s8qqaq=dirpe0xb9q8qiLsFr0=vr0=vr0dc8meaabaqaciaacaGaaeqabaqabeGadaaakeaacqWGubavcqGGOaakcqWG0baDcqGGPaqkcqGH9aqpcqWGRbWAiiGacqWF4oqCcqGGOaakcqWG0baDcqGGPaqkcqGHRaWkcqWF3oaAdaWcaaqaaiabdsgaKjab=H7aXjabcIcaOiabdsha0jabcMcaPaqaaiabdsgaKjabdsha0baacqGHRaWkcqWGjbqsdaWcaaqaaiabdsgaKnaaCaaaleqabaGaeGOmaidaaOGae8hUdeNaeiikaGIaemiDaqNaeiykaKcabaGaemizaqMaemiDaq3aaWbaaSqabeaacqaIYaGmaaaaaOGaaCzcaiaaxMaadaqadaqaaiabigdaXaGaayjkaiaawMcaaaaa@54C8@

where *T(t) *is the forcing torque, *θ(t) *is the angular position, *k *is the constant for the torsional elastic recoil, *η *is the constant for the torsional viscous dissipation and *I *is the moment of inertia of the oscillating mass with respect to its axis of rotation.

When forced by a sinusoidal input of frequency *f*, the input-output phase relation can be expressed by the two coefficient equation:

Φ(f)=−arctanγfπ(fr2−f2)     (2)
 MathType@MTEF@5@5@+=feaafiart1ev1aaatCvAUfKttLearuWrP9MDH5MBPbIqV92AaeXatLxBI9gBaebbnrfifHhDYfgasaacH8akY=wiFfYdH8Gipec8Eeeu0xXdbba9frFj0=OqFfea0dXdd9vqai=hGuQ8kuc9pgc9s8qqaq=dirpe0xb9q8qiLsFr0=vr0=vr0dc8meaabaqaciaacaGaaeqabaqabeGadaaakeaacqqHMoGrcqGGOaakcqWGMbGzcqGGPaqkcqGH9aqpcqGHsislcqWGHbqycqWGYbGCcqWGJbWycqWG0baDcqWGHbqycqWGUbGBdaWcaaqaaGGaciab=n7aNHqaciab+zgaMbqaaiab=b8aWnaabmaabaGaemOzay2aa0baaSqaaiabdkhaYbqaaiabikdaYaaakiabgkHiTiabdAgaMnaaCaaaleqabaGaeGOmaidaaaGccaGLOaGaayzkaaaaaiaaxMaacaWLjaWaaeWaaeaacqaIYaGmaiaawIcacaGLPaaaaaa@4CB7@

where Φ(*f*) is the input-output phase, ⤢fr=12πkI⥄⥄
 MathType@MTEF@5@5@+=feaafiart1ev1aaatCvAUfKttLearuWrP9MDH5MBPbIqV92AaeXatLxBI9gBaebbnrfifHhDYfgasaacH8akY=wiFfYdH8Gipec8Eeeu0xXdbba9frFj0=OqFfea0dXdd9vqai=hGuQ8kuc9pgc9s8qqaq=dirpe0xb9q8qiLsFr0=vr0=vr0dc8meaabaqaciaacaGaaeqabaqabeGadaaakeaacaaMfSUaemOzay2aaSbaaSqaaiabdkhaYbqabaGccqGH9aqpdaWcaaqaaiabigdaXaqaaiabikdaYGGaciab=b8aWbaadaGcaaqaamaalaaabaGaem4AaSgabaGaemysaKeaaaWcbeaakiaaykW6caaMcSoaaa@3BA7@ is the resonance frequency and γ=η2I
 MathType@MTEF@5@5@+=feaafiart1ev1aaatCvAUfKttLearuWrP9MDH5MBPbIqV92AaeXatLxBI9gBaebbnrfifHhDYfgasaacH8akY=wiFfYdH8Gipec8Eeeu0xXdbba9frFj0=OqFfea0dXdd9vqai=hGuQ8kuc9pgc9s8qqaq=dirpe0xb9q8qiLsFr0=vr0=vr0dc8meaabaqaciaacaGaaeqabaqabeGadaaakeaaiiGacqWFZoWzcqGH9aqpdaWcaaqaaiab=D7aObqaaiabikdaYiabdMeajbaaaaa@3324@ is the damping coefficient. The phase response resulting from Eq. (2) starts from 0° at *f *= 0 Hz and decays sigmoidally to -180° for *f*→∞.

The experimental input-output phase difference between the MnI and movement sinusoids was measured (*mech *data) between two discrete corresponding points (see the above paragraph *Measurements of the relevant phase delays *and Fig. 1) where both the value of the active torque (zero) and the limb position are precisely defined. Thus, values plotted in the *mech *curves give real torque-to-position phase delays.

Fitting of the limb phase-response (*mech *experimental data, see above) with Eq. (2) by a least square procedure assessed the model adequacy in describing the segment oscillations and provided parametrical values for statistical comparisons (see below).

### Statistical analysis

A two-way repeated measures ANOVA was used to evaluate the effects of limb coupling and oscillation frequency on the *clk-mov *curves. Results are reported in Table 1.

Data of the *mech *curves from all subjects were first fitted by the pendulum equation (Eq. 2) to evaluate the two descriptive parameters *f*_*r *_(resonance frequency) and *γ *(damping coefficient). Best fit functions were estimated by the least squares method and the quality of fitting was expressed by the *R*^2 ^coefficient. The reliability of the asymptotic standard error estimates resulting from the fitting procedure rests on the Gaussian distribution of the two parameters, ascertained by the Kolmogorov-Smirnov test for continuous data applied on the result of a Monte Carlo simulation on Eq. (2) (cfr.) [43].

The *mech *curves obtained in each limb in the uncoupled and coupled conditions were compared with each other by matching the parameters (*f*_*r *_and *γ*) of their best-fit functions through the extra sum-of-squares F test (cfr.) [44] This procedure was justified by the strict correspondence of the model to the data points, as witnessed by the high values of *R*^2^. Results of these calculations are reported in table 2. A comparison was also drawn between the *mech *curves of the unloaded hand and foot. Since in this case the F test revealed a significant difference between the curves, the effect of each parameter was separately evaluated by restricting the F test to that parameter only (cfr.) [44].

Since the *neur *delay derives from the subtraction of the *mech *from the *clk-mov *intervals, a comparison between the *neur *curves was unnecessary. It would in fact re-test the same samples used for comparing the *clk-mov *and *mech *curves, causing the type I error probability to rise.

Finally, a three way repeated measures ANOVA, followed by a Newman-Keuls post-hoc test, evaluated the effect of frequency, loading and association type (iso- or antidirectional) on the inter-limb phase differences measured in the various loading and association combinations. In all statistical tests, significance level was set at 0.05.

## Authors' contributions

FGB conceived and co-ordinated the study, acquired the funding, acquired and interpreted the data, composed the manuscript. PC participated in the acquisition and interpretation of data, revised the manuscript. RE conceived the study, acquired and interpreted the data, performed the statistical analysis, drafted the manuscript. All authors read and approved the final manuscript.

## Figures and Tables

**Table 1:** Statistical comparison between the *clk-mov *curves of the hand and foot.

	segment	coupled with	coupling main effect	frequency main effect	interaction
isodirectional association	hand unloaded	alone			
		foot unloaded	F_(2,18) _= 0.32	F_(8,72) _= 1.25	F_(16,144) _= 1.20
		foot loaded	p = 0.732	p = 0.284	p = 0.274
	
	foot unloaded	alone			
		hand unloaded	F_(2,18) _= 1.80	F_(8,72) _= 1.98	F_(16,144) _= 1.19
		hand loaded	p = 0.194	p = 0.060	p = 0.278
	
	hand loaded	alone	F_(1,9) _= 1.61	F_(8,72) _= 3.02	F_(8,72) _= 1.02
		foot unloaded	p = 0.236	p < 0.0055*	p = 0.426
	
	foot loaded	alone	F_(1,9) _= 2.66	F_(8,72) _= 7.13	F_(8,72) _= 0.49
		hand unloaded	p = 0.137	p < 0.000001*	p = 0.856

antidirectional association	hand unloaded	alone			
		foot unloaded	F_(2,18) _= 0.16	F_(5,45) _= 1.94	F_(10,90) _= 0.68
		foot loaded	p = 0.854	p = 0.105	p = 0.739
	
	foot unloaded	alone			
		hand unloaded	F_(2,18) _= 2.17	F_(5,45) _= 1.04	F_(10,90) _= 0.96
		hand loaded	p = 0.143	p = 0.406	p = 0.481
	
	hand loaded	alone	F_(1,9) _= 1.71	F_(5,45) _= 11.03	F_(5,45) _= 1.06
		foot unloaded	p = 0.224	p = 0.000001*	p = 0.394
	
	foot loaded	alone	F_(1,9) _= 1.93	F_(5,45) _= 8.06	F_(5,45) _= 1.33
		hand unloaded	p = 0.198	p = 0.00002*	p = 0.270

Two-way repeated measures ANOVA, 2 or 3 coupling modalities × 9 frequencies. Direction of movement association is indicated in col.1, moving segment in col.2 and coupling modality in col.3. Significant effects marked by asterisk. In all the configurations examined, the ANOVA test failed to reveal any significant effect of coupling on the *clk-mov *curves. The *p *values were very high in two configurations and much lower (0.137 <*p *< 0.236) in the others. It is worth noting, however, that in all the latter cases, the effect of coupling, even if not significant, was opposite to that expected, i.e. the lagging segment was phase-delayed or the leading segment was phase-advanced.

**Table 2:** Statistical comparison between the descriptive parameters of the *mech *curves of the hand and foot.

	segment	coupled with	*f*_*r *_(Hz)	*γ *(s^-1^)	*R*^2^	comparison
isodirectional association	hand unloaded	alone	2.542 ± 0.048	6.882 ± 0.623	0.874	
		foot unloaded	2.496 ± 0.054	7.305 ± 0.716	0.856	F_(4,264) _= 0.25
		foot loaded	2.517 ± 0.064	7.060 ± 0.838	0.815	p = 0.91
	
	foot unloaded	alone	3.272 ± 0.124	12.510 ± 1.701	0.830	
		hand unloaded	3.320 ± 0.118	12.650 ± 1.594	0.855	F_(4,264) _= 0.24
		hand loaded	3.193 ± 0.118	11.000 ± 1.561	0.810	p = 0.92
	
	hand loaded	alone	1.314 ± 0.041	3.332 ± 0.340	0.872	F_(2,176) _= 0.40
		foot unloaded	1.279 ± 0.044	2.937 ± 0.346	0.845	p = 0.67
	
	foot loaded	alone	1.464 ± 0.045	2.549 ± 0.344	0.851	F_(2,176) _= 0.22
		hand unloaded	1.480 ± 0.044	2.852 ± 0.342	0.864	p = 0.80

antidirectional association	hand unloaded	alone	2.206 ± 0.168	4.023 ± 1.322	0.500	
		foot unloaded	2.630 ± 0.370	6.977 ± 2.975	0.489	F_(4,174) _= 0.74
		foot loaded	2.638 ± 0.487	8.298 ± 4.485	0.404	p = 0.56
	
	foot unloaded	alone	5.113 ± 3.396	47.72 ± 67.82	0.550	
		hand unloaded	5.648 ± 5.649	50.68 ± 107.4	0.458	F_(4,174) _= 0.55
		hand loaded	3.449 ± 1.363	18.03 ± 16.99	0.343	p = 0.70
	
	hand loaded	alone	1.213 ± 0.046	3.635 ± 0.506	0.766	F_(2,116) _= 1.43
		foot unloaded	1.138 ± 0.040	2.687 ± 0.379	0.799	p = 0.24
	
	foot loaded	alone	1.441 ± 0.065	4.876 ± 0.828	0.691	F_(2,116) _= 0.12
		hand unloaded	1.407 ± 0.058	4.396 ± 0.689	0.719	p = 0.89

Comparison was drawn trough the extra sum-of-squares F test on *f*_*r *_(resonance frequency) and *γ *(damping coefficient). Direction of movement association is indicated in col.1, moving segment in col. 2, coupling modality in col. 3. Col. 4 and 5: mean values ± SE of *f*_*r *_and *γ *. Col. 6: coefficient of correlation *R*^2 ^with the pendulum equation. Col. 7: F and p values of the comparisons.
